# Beta-blockers after myocardial infarction with preserved or mildly reduced ejection fraction: existing evidence, knowledge gaps, and an EF-stratified framework

**DOI:** 10.3389/fphar.2026.1821921

**Published:** 2026-04-21

**Authors:** Shujuan Zhao, Chiwei Guo, Haixia Cai, Lei Wang

**Affiliations:** Department of Pharmacy, Henan Provincial People’s Hospital, People’s Hospital of Zhengzhou University, School of Clinical Medicine, Henan University, Zhengzhou, Henan, China

**Keywords:** beta-blockers, guideline-directed medical therapy, LVEF, myocardial infarction, secondary prevention

## Abstract

**Background:**

For several decades, beta-blockers (BBs) have served as a cornerstone in the secondary prevention following myocardial infarction (MI). However, contemporary randomized evidence has challenged the long-term BB use in patients with preserved or mildly reduced left ventricular ejection fraction (LVEF) in the era of percutaneous coronary intervention and comprehensive guideline-directed medical therapy.

**Methods:**

We summarized evidence from three randomized controlled trials and two contemporary meta-analyses, evaluating BB therapy in post-MI patients with preserved or mildly reduced LVEF. We critically appraised trial designs, endpoints, and outcomes stratified by LVEF, and reviewed current guideline recommendations from North American and European perspectives.

**Results:**

In patients with preserved LVEF (≥50%), pooled data demonstrated no reduction in death, MI, or heart failure with BB therapy (*P* = 0.54). By contrast, among patients with mildly reduced LVEF (40%–49%), BB was associated with a 25% relative risk reduction in the composite endpoint (*P* = 0.031), with no between-trial heterogeneity. Safety profiles were comparable between BB and no-BB groups across all trials.

**Conclusion:**

Contemporary evidence supports an EF-stratified approach to BB therapy after MI. Routine long-term BB prescription may no longer be justified for patients with preserved LVEF (≥50%) without other indications, whereas BB therapy may be reasonable to consider for those with mildly reduced LVEF (40%–49%). These findings support an EF-stratified approach to long-term BB use after MI in contemporary practice.

## Introduction

For nearly half a century, beta-adrenergic receptor antagonists (beta-blockers, BBs) have served as a cornerstone in secondary prevention following myocardial infarction (MI) and the treatment of heart failure (HF). Historically, BB efficacy was established in the pre-reperfusion era, when residual ischemia was common, and disease-modifying adjunctive therapies were limited (Johner et al., 2024; Cataldo Miranda et al., 2025). Mechanistically, BBs antagonize sympathetic nervous activation, thereby reducing myocardial oxygen demand, preventing maladaptive ventricular remodeling, and increasing the threshold for malignant arrhythmias (Johner et al., 2024).

However, cardiovascular medicine has undergone a profound transformation. The widespread use of mechanical reperfusion strategies, specifically percutaneous coronary intervention (PCI), coupled with introduction of potent antithrombotic regimens, and high-intensity statins, have substantially improved outcomes after MI (Yndigegn et al., 2024; Johner and Gencer, 2025). Consequently, the absolute risk reduction conferred by BB therapy in this contemporary context has been increasingly questioned (Supplementary Table S1).

Current international guidelines categorize HF into three phenotypes (McDonagh et al., 2021; McDonagh et al., 2023; Heidenreich et al., 2022): HF with reduced ejection fraction (HFrEF, left ventricular ejection fraction [LVEF] ≤ 40%), HF with mildly reduced ejection fraction (HFmrEF, LVEF 41%–49%), and HF with preserved ejection fraction (HFpEF, LVEF ≥ 50%). While BBs remain foundational in HFrEF, the evidence for their use in post-MI patients with preserved or mildly reduced LVEF is uncertain.

Accordingly, this article summarizes the evolving evidence, provides an update of contemporary randomized controlled trials (RCTs), meta-analyses, and guideline positions, highlights knowledge gaps, and proposes an ejection fraction (EF)-stratified framework to guide BB use after MI in patients with preserved or mildly reduced LVEF. It is important to note that the clinical question of post-MI BB therapy encompasses three distinct scenarios: acute initiation during hospitalization, long-term continuation as secondary prevention, and withdrawal of established therapy. These scenarios carry different evidence bases and clinical implications. The present perspective primarily addresses long-term continuation and potential withdrawal in hemodynamically stable patients.

## Evidence selection

**FIGURE 1:**
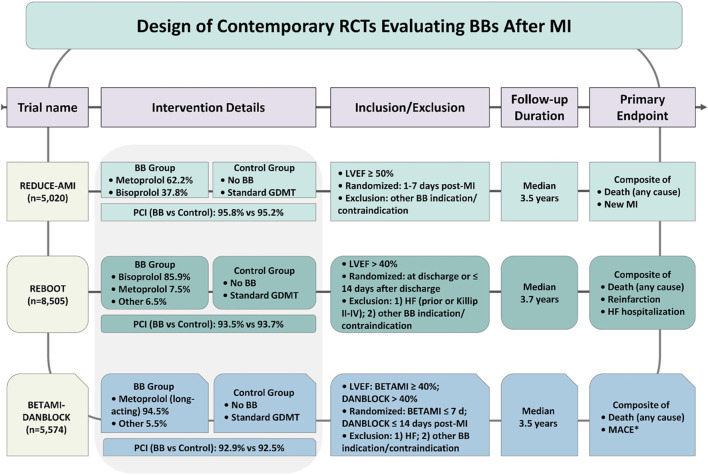
Design of contemporary randomized controlled trials evaluating BBs after myocardial infarction. Abbreviations: RCT, randomized controlled trial; BB, beta-blocker; MI, myocardial infarction; PCI, percutaneous coronary intervention; GDMT, guideline-directed medical therapy; LVEF, left ventricular ejection fraction; HF, heart failure; MACE, major adverse cardiovascular event. *MACE includes new myocardial infarction, unplanned coronary revascularization, ischemic stroke, heart failure, and malignant ventricular arrhythmias.

We focused on contemporary RCTs that met three criteria: (1) randomized comparison of BB therapy versus no BB (or placebo) after acute MI; (2) enrollment restricted to patients with preserved or mildly reduced LVEF (i.e., LVEF ≥ 40% or ≥ 50%); and (3) conducted in the modern era of routine PCI and comprehensive guideline-directed medical therapy (GDMT). Three trials met these criteria (Figure 1): the REDUCE-AMI trial (Randomized Evaluation of Decreased Usage of Beta-Blockers after Acute Myocardial Infarction) (Yndigegn et al., 2024), the REBOOT trial (Treatment with Beta-Blockers after Myocardial Infarction without Reduced Ejection Fraction) (Ibanez et al., 2025), and the BETAMI-DANBLOCK trials (Norwegian Beta-Blocker Treatment after Acute Myocardial Infarction in Revascularized Patients without Reduced Left Ventricular Ejection Fraction/Danish Trial of Beta-Blocker Therapy after Myocardial Infarction without Heart Failure) (Munkhaugen et al., 2025). We further included two recent individual-patient-data or aggregate-data meta-analyses that pooled these trials stratified by LVEF (Kristensen et al., 2025; Rossello et al., 2025).

To contextualize these findings within current clinical practice, we summarized the latest guideline documents, including the 2025 ACC/AHA Guideline for the Management of Patients With Acute Coronary Syndromes (Rao et al., 2025), the 2023 ESC Guidelines for the Management of Acute Coronary Syndromes (Byrne et al., 2023), the 2023 AHA/ACC Guideline for the Management of Patients With Chronic Coronary Disease (Virani et al., 2023), and the 2024 ESC Guidelines for the Management of Chronic Coronary Syndromes (Vrints et al., 2024). Given the focused scope of this perspective article, we did not perform a formal systematic search. Instead, evidence selection was curated to reflect the most current and clinically relevant standards of care.

## Contemporary RCTs

### REDUCE-AMI trial

The REDUCE-AMI trial (Yndigegn et al., 2024) was a prospective, open-label, parallel-group RCT. It enrolled patients with MI and preserved LVEF (≥50%). All participants underwent coronary angiography and echocardiography, and obstructive coronary artery disease (CAD) was required for inclusion. REDUCE-AMI enrolled 5,020 patient (35.0% in the BB group and 35.5% in the no-BB group had ST-Elevation Myocardial Infarction [STEMI], and the remaining patients had Non-ST-Elevation Myocardial Infarction [NSTEMI]), and the median follow-up was 3.5 years. Coronary revascularization was common during the index hospitalization, with PCI performed in 95.8% of the BB group and 95.2% of the no-BB group. The primary endpoint was a composite of all-cause death or new MI. The primary endpoint event occurred in 199/2,508 (7.9%) patients assigned to BBs and 208/2,512 (8.3%) assigned to no-BBs (hazard ratio [HR] 0.96, 95% confidence interval [CI] 0.79–1.16; *P* = 0.64). Secondary outcomes, including all-cause death, recurrent MI, and hospitalization for HF, were also similar between groups, and safety endpoints did not differ materially (Supplementary Table S2).

The REDUCE-AMI trial, which evaluated long-term BB continuation as secondary prevention, provides contemporary evidence that routine long-term BB therapy did not improve hard clinical outcomes in post-MI patients with preserved LVEF. However, questions remain regarding potential benefits in selected subgroups and in patients with mildly reduced LVEF.

### REBOOT trial

The REBOOT trial (Ibanez et al., 2025) was a prospective, randomized, open-label, blinded-endpoint RCT. Patients with acute MI were eligible if they received invasive care during the index hospitalization and had LVEF > 40% before discharge. Among 8,505 randomized patients, 8,438 were included in the main analysis (4,207 BB vs. 4,231 no BB). Of these, 51.0% of patients in the BB group and 50.8% in the no-BB group had STEMI, while 49.0% in the BB group and 49.2% in the no-BB group had NSTEMI. The median follow-up was 3.7 years. PCI was common (93.5% in the BB group vs. 93.7% in the no-BB group). The primary endpoint was a composite of all-cause death, reinfarction, or HF hospitalization. A primary endpoint event occurred in 316 patients in BB group and 307 in no-BB group (HR 1.04, 95% CI 0.89–1.22; *P* = 0.63). The individual components were also similar: all-cause death (161 vs. 153; HR 1.06), reinfarction (143 vs. 143; HR 1.01), and HF hospitalization (39 vs. 44; HR 0.89). No apparent between-group differences in safety outcomes were noted (Supplementary Table S2).

Overall, the REBOOT trial provides randomized evidence on long-term BB continuation in patients with LVEF > 40%. The trial showed that routine BB therapy initiated around discharge did not reduce the composite endpoint of all-cause death, reinfarction, or HF hospitalization after MI in the setting of modern invasive care and GDMT.

### BETAMI–DANBLOCK trials

The BETAMI–DANBLOCK trials (Munkhaugen et al., 2025) were open-label, parallel-group RCTs with blinded endpoint evaluation conducted in Norway (BETAMI) and Denmark (DANBLOCK). Patients were eligible if they provided informed consent within 7 days (BETAMI) or 14 days (DANBLOCK) after MI, and had LVEF ≥ 40% (BETAMI) or LVEF > 40% (DANBLOCK); BETAMI required coronary revascularization, whereas DANBLOCK did not. 5,574 patients were included in the main analyses (2,783 BB vs. 2,791 no BB), with a median follow-up of 3.5 years. 47.8% of patients in the BB group and 47.2% of patients in the no-BB group had STEMI, and the remaining patients had NSTEMI. Contemporary care was common, with PCI in 92.9% vs. 92.5% (BB vs. no BB).

The primary endpoint was a composite of all-cause death or major adverse cardiovascular events (MACEs): new MI, unplanned coronary revascularization, ischemic stroke, HF, or malignant ventricular arrhythmias (MVA). A primary endpoint event occurred in 394/2,783 (14.2%) patients in the BB group and 454/2,791 (16.3%) in the no-BB group (HR 0.85, 95% CI 0.75–0.98; *P* = 0.03). All-cause death was similar between groups (4.2% vs. 4.4%), whereas new MI occurred less frequently with BB therapy (5.0% vs. 6.7%; HR 0.73, 95% CI 0.59–0.92). No apparent between-group differences in safety outcomes were observed (Supplementary Table S2).

Overall, BETAMI–DANBLOCK suggests that, among patients with MI and LVEF ≥ 40% (BETAMI)/> 40% (DANBLOCK), a long-term BB strategy was associated with a modest reduction in composite endpoint, mainly driven by fewer new MI events, without a clear mortality difference.

## Meta-analyses Re-evaluating BBs efficacy

### Mildly reduced LVEF (40%–49%)

**FIGURE 2:**
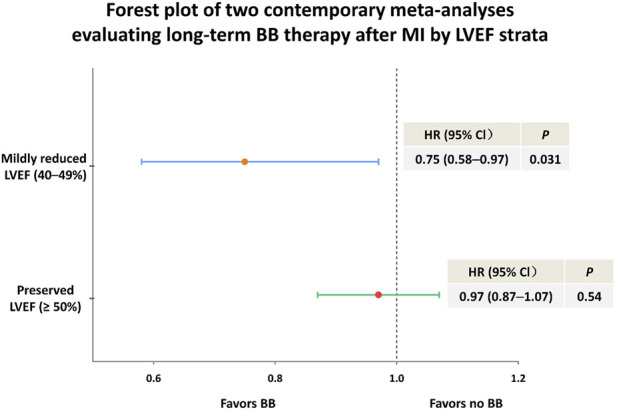
Forest plot of two contemporary meta-analyses evaluating long-term BB therapy after MI by LVEF strata. Abbreviations: BB, beta-blocker; MI, myocardial infarction; LVEF, left ventricular ejection fraction; HR, hazard ratio; CI, confidence interval.

A meta-analysis pooled patients with mildly reduced LVEF (40%–49%) (Figure 2) (Rossello et al., 2025). The trials evaluated long-term oral BB therapy initiated within 14 days after acute MI. Eligible patients had no history or clinical signs of HF. The prespecified primary composite endpoint was all-cause death, new MI, or HF. Overall, 1,885 patients were included. A total of 991 were assigned to BBs and 894 to control (no BBs), with a median follow-up of 3.5 years. The primary composite endpoint occurred in 106/991 patients in BB group (32.6 events per 1,000 patient-years) and in 129/894 patients in control group (43.0 events per 1,000 patient-years), corresponding to an HR of 0.75 (95% CI 0.58–0.97; *P* = 0.031). There was no evidence of heterogeneity across trials (*P* for interaction = 0.95) (Table 1) (Rossello et al., 2025). Sensitivity analyses were consistent. In the LVEF 41%–49% subgroup, the HR was 0.73 (95% CI 0.54–0.98; *P* = 0.037). All-cause death and new MI showed numerically lower event rates with BBs, but were not statistically significant when assessed individually (Table 1) (Rossello et al., 2025).

**TABLE 1:** Summary of two meta-analyses evaluating long-term beta-blocker therapy after myocardial infarction.

Item	Meta-analysis (4 RCTs; LVEF 40%–49%)	Meta-analysis (5 RCTs; LVEF ≥ 50%)
LVEF stratum	Mildly reduced LVEF (40%–49%)	Preserved LVEF (≥50%)
Included trials	REBOOT, BETAMI, DANBLOCK, CAPITAL-RCT	REBOOT, REDUCE-AMI, BETAMI, DANBLOCK, CAPITAL-RCT
Key population/criteria	Acute MI; BB started ≤ 14 days; no history/clinical signs of HF	Recent MI; no other BB indications; randomized to BB vs. no BB
Sample size	N = 1,885 (BB 991 vs. no BB 894)	N = 17,801 (BB 8,831 vs. no BB 8,970)
Follow-up	Median 3.5 years	Median 3.6 years
Primary composite endpoint	All-cause death, new MI, or HF	Death (any cause), MI, or HF
Primary result	106/991 (32.6/1000 PY) vs. 129/894 (43.0/1000 PY); **HR 0.75 (95% CI 0.58–0.97); *P* = 0.031**; no heterogeneity (*P* for interaction = 0.95)	717/8,831 (8.1%) vs. 748/8,970 (8.3%); **HR 0.97 (95% CI 0.87–1.07); *P* = 0.54**
Sensitivity/components	LVEF 41%–49%: HR 0.73 (95% CI 0.54–0.98); *P* = 0.037. Death/MI numerically lower but not significant individually	Components: death HR 1.04 (95% CI 0.89–1.21); MI HR 0.89 (95% CI 0.77–1.03); HF HR 0.87 (95% CI 0.64–1.19)
Safety	Stroke numerically higher with BBs (13 vs. 7 events; not significant)	Ischemic stroke: 115 vs. 94; reported as **RMST difference at 3 years = 2.6 days (95% CI −0.73 to 4.4)** (not HR). Advanced atrioventricular block*: 69/8, 510 vs. 68/8, 634; HR 1.03 (95% CI 0.73–1.44)
Summary	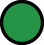 **Benefit signal** (mildly reduced LVEF)	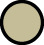 **Neutral** (preserved LVEF)

Abbreviations: RCT, randomized controlled trial; LVEF, left ventricular ejection fraction; MI, myocardial infarction; BB, beta-blocker; HF, heart failure; HR, hazard ratio; CI, confidence interval; PY, patient-years; RMST, restricted mean survival time.

* Denominators reflect available safety data.

Traffic-light summary: green = benefit signal; gray = neutral.

Bold values indicate the primary effect estimates and key statistical results for each meta-analysis.

### Preserved LVEF (≥50%)

Another meta-analysis pooled patients with preserved LVEF (≥50%) (Figure 2) (Kristensen et al., 2025). 17,801 patients were included (8,831 assigned to BBs and 8,970 to no BBs). Median follow-up was 3.6 years. The prespecified primary composite endpoint was death from any cause, MI, or HF. A primary endpoint event occurred in 717/8,831 (8.1%) in BB group and 748/8,970 (8.3%) in no-BB group (HR 0.97, 95% CI 0.87–1.07; *P* = 0.54) (Table 1) (Kristensen et al., 2025). The individual components were also similar between groups. Death from any cause occurred in 335 versus 326 patients (HR 1.04, 95% CI 0.89–1.21). MI occurred in 360 versus 407 patients (HR 0.89, 95% CI 0.77–1.03). HF occurred in 75 versus 87 patients (HR 0.87, 95% CI 0.64–1.19) (Table 1) (Kristensen et al., 2025). For safety endpoint, ischemic stroke occurred in 115 (BB group) vs. 94 patients (no-BB group). It was reported as a restricted mean survival time (RMST) difference at 3 years (2.6 days; 95% CI −0.73 to 4.4), instead of a hazard ratio. Advanced atrioventricular block occurred in 69 vs. 68 patients in the two groups (HR 1.03, 95% CI 0.73–1.44) (Kristensen et al., 2025).

## Contemporary guidelines

### North American perspective

**FIGURE 3:**
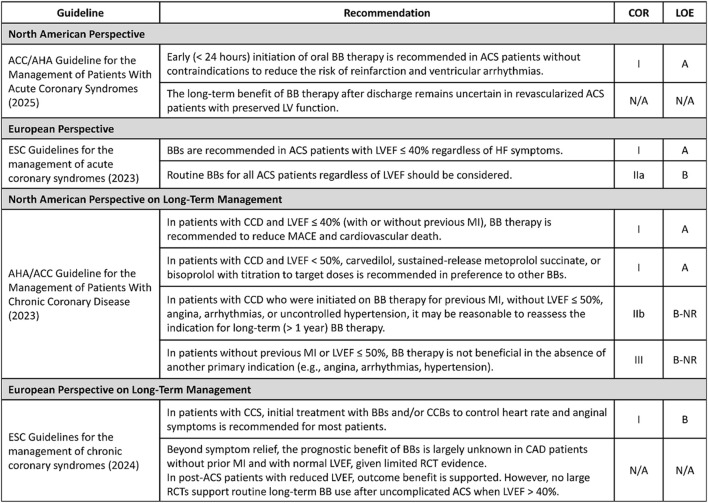
Contemporary guideline recommendations for the use of BB in patients after ACS and MI. Abbreviations: BB, beta-blocker; ACS, acute coronary syndrome; MI, myocardial infarction; ACC, American College of Cardiology; AHA, American Heart Association; ESC, European Society of Cardiology; LV, left ventricle; COR, class of recommendation; LOE, level of evidence; N/A, not applicable; LVEF, left ventricular ejection fraction; CCD, chronic coronary disease; CCS, chronic coronary syndrome; CCB, calcium-channel blocker; CAD, coronary artery disease; MI, myocardial infarction; RCT, randomized controlled trial.

The North American guideline emphasizes early initiation of oral BB therapy in patients without contraindications (Figure 3). Specifically, a low dose of oral BB should be initiated early (<24 h) after diagnosis of acute coronary syndrome (ACS), with slow dose escalation as blood pressure and heart rate allow. It also advises that BBs should be discontinued in patients who develop new or worsening HF symptoms or signs of cardiogenic shock (Rao et al., 2025). However, the current guideline acknowledges that, among patients with preserved LVEF, the optimal duration of BB therapy after discharge remains uncertain.

### European perspective

The 2023 European Society of Cardiology (ESC) Acute Coronary Syndrome guideline supports BB therapy as part of long-term management after ACS, with the strongest emphasis in patients with reduced systolic function (Figure 3). Specifically, BBs are recommended for ACS patients with LVEF ≤ 40%, regardless of HF symptoms. For the broader ACS population, the guideline notes that routine BB therapy for all ACS patients, regardless of LVEF, should be considered (Byrne et al., 2023). Overall, the ESC guidance supports BB therapy across the ACS population, with the clearest recommendation in patients with LVEF ≤ 40%.

### North American perspective long-term management

The 2023 AHA/ACC Chronic Coronary Disease (CCD) guideline provides complementary long-term recommendations (Figure 3) (Virani et al., 2023). For patients with CCD and LVEF ≤ 40%, BB therapy is recommended to reduce MACE and cardiovascular death (Class I). In patients with CCD and LVEF < 50%, carvedilol, sustained-release metoprolol succinate, or bisoprolol is recommended in preference to other BBs (Class I). Notably, for patients initiated on BB therapy for previous MI without LVEF ≤ 50%, angina, arrhythmias, or uncontrolled hypertension, the guideline states that it may be reasonable to reassess the indication for long-term (>1 year) BB therapy (Class IIb). In patients with CCD without prior MI or LVEF ≤ 50%, BB therapy is not beneficial for reducing cardiovascular events in the absence of another primary indication (Class III).

### European perspective on long-term management

The 2024 ESC Chronic Coronary Syndromes (CCS) guideline recommends BBs primarily as antianginal therapy in the long-term phase (Figure 3) (Vrints et al., 2024). It notes that, for many CCS patients, initial symptom-based medical therapy can include a BB and/or a CCB, with treatment tailored to haemodynamics and comorbidities. For prognosis, it states that in CAD patients without prior MI and with preserved LVEF, the prognostic benefit of BBs is largely unknown. By contrast, outcome benefits in post-ACS patients with reduced LVEF are supported by well-established evidence. Overall, both North American and European long-term guidelines converge on the principle that BB therapy is well-supported in patients with reduced LVEF, whereas its role in patients with preserved LVEF after MI remains uncertain.

## Critical discussions of updated evidence

A recent editorial accompanying the Kristensen et al. meta-analysis provided a timely overview and underscored the need for personalized management in the post-MI setting (Lüscher and Wenzl, 2026). The present perspective extends this discussion by providing detailed trial-level comparisons, examining biological plausibility, addressing the practical question of BB discontinuation, and proposing an explicit EF-stratified framework.

## LVEF-stratified management

The contemporary randomized evidence reveals a gradient effect between BB efficacy and LVEF. In patients with preserved LVEF (≥50%) (Kristensen et al., 2025), evidence from 17,801 patients of a meta-analysis demonstrates a consistent neutral signal for the composite of death, MI, or HF, establishing the highest level of negative evidence for routine BB therapy in this population. In contrast, the individual data meta-analysis specifically isolated 1,885 patients with mildly reduced LVEF (40%–49%). This analysis revealed a significant 25% relative risk reduction in the composite endpoint of all-cause death, new MI, or HF (Rossello et al., 2025). This represents a pivotal finding, the mildly reduced LVEF range (40%–49%) has historically occupied an ambiguous therapeutic position, often receiving extrapolated recommendations from HFrEF trials without dedicated evidence (Zafeiropoulos et al., 2024). The clinical implications of this EF-stratified framework are substantial: BBs may be reasonable for LVEF 40%–49%, while the evidence no longer supports routine use for LVEF ≥ 50% in the absence of other compelling indications, although further confirmation from dedicated trials is needed.

## Why BETAMI-DANBLOCK differed

The discrepancy between the positive findings of BETAMI–DANBLOCK (Munkhaugen et al., 2025) and the neutral results of REDUCE-AMI (Yndigegn et al., 2024) and REBOOT (Ibanez et al., 2025) has generated considerable debate. However, closer examination reveals that these results are more consistent than they initially appear.

The benefit observed in BETAMI–DANBLOCK trials was almost entirely driven by a reduction of new MI events (5.0% vs. 6.7%; HR 0.73, 95% CI 0.59–0.92) (Munkhaugen et al., 2025), with approximately 80% of these new MI events being NSTEMIs, which were typically considered smaller infarctions in patients who had already undergone revascularization (Bhatt et al., 2022). Importantly, no differences were observed in all-cause mortality, HF hospitalization, or MVA. The broader six-component composite endpoint, including unplanned coronary revascularization, ischemic stroke, and MVA, introduced additional heterogeneity and susceptibility to surveillance bias.

Overall, the modest benefit from BETAMI–DANBLOCK trial may reflect the early anti-ischemic effects of BB rather than durable hard-endpoint protection (Munkhaugen et al., 2025). When the analysis is restricted to the core hard endpoints of death and HF, results across all three contemporary trials converge toward a consistent neutral signal.

## Biological plausibility

The landmark trials that established BB therapy after MI were conducted when the therapeutic targets of BB, such as large infarct size, residual ischemia, and MVA, were prevalent (Kim et al., 2022; Cataldo Miranda et al., 2025). In the pre-reperfusion era, patients who survived the acute phase of MI often had substantial myocardial necrosis, adverse ventricular remodeling, and heightened sympathetic activation, creating optimal foundation for BB-mediated protection (Hong and Barry, 2018). The reduction in sudden cardiac death observed in trials such as the Norwegian Timolol Study reflected the properties of BB in this high-risk population (Norwegian Multicenter Study Group, 1981; β-Blocker Heart Attack Trial Research Group, 1982; Hjalmarson et al., 1983).

Contemporary primary PCI has altered this landscape. Timely reperfusion limits infarct size, preserves myocardial function, and substantially reduces the incidence of life-threatening ventricular arrhythmias (Mignatti et al., 2025). In patients who achieve complete revascularization and maintain LVEF ≥ 50%, the pathophysiological problem that BB was designed to address, including electrical instability arising from large areas of scarred, heterogeneous myocardium, is largely absent. The arrhythmic risk in this population is low, leaving minimal room for BB to confer additional protection (Sabina et al., 2024; Chi et al., 2025). The contemporary pharmacological environment has further attenuated the potential for BB to demonstrate benefit. Modern GDMT includes high-intensity statins and dual antiplatelet therapy (DAPT), each of which independently reduces cardiovascular event risk (Cotter et al., 2024; Tang et al., 2024). The cumulative effect of these therapies has substantially lowered the baseline absolute risk in post-MI patients with preserved LVEF.

Patients with mildly reduced LVEF (40%–49%) represent an intermediate phenotype characterized by borderline contractile dysfunction and potentially subclinical neurohormonal activation (Desai et al., 2024). This population may have residual adverse remodeling and arrhythmogenic risk that remains responsive to the sympatholytic and anti-remodeling effects of BB therapy (Savarese et al., 2022). The 25% relative risk reduction observed in the meta-analysis suggests the existence of a critical threshold of myocardial vulnerability (Rossello et al., 2025). Below this threshold, BB therapy retains clinical relevance.

## Evolution of guidelines

The Class I recommendation for routine BB therapy in all post-MI patients was established during an era when the therapeutic landscape differed from contemporary practice. The landmark trials that formed the evidentiary foundation were conducted before primary PCI, high-intensity statins, and modern DAPT (Norwegian Multicenter Study Group, 1981; β-Blocker Heart Attack Trial Research Group, 1982). While BB demonstrated unequivocal mortality reduction in that context, these recommendations persisted largely unchanged for decades, even as the patient population and standard of care underwent transformation.

Recent guideline updates reflect growing recognition of this evidence gap. The 2023 ESC Guidelines acknowledged that “the role of long-term BB therapy in patients without HF or left ventricle systolic dysfunction remains uncertain” (Byrne et al., 2023). Similarly, the 2025 North American Guidelines indicated that “may be reasonable to reassess” BB continuation in patients with preserved LVEF (Rao et al., 2025). The 2023 AHA/ACC CCD guideline had already introduced a Class IIb recommendation for reassessing long-term BB therapy in post-MI patients without LVEF ≤ 50% or other compelling indications (Virani et al., 2023). This shift represents an evolving stance among guideline committees. Notably, both European and American guidelines are now moving toward a more individualized, EF-stratified approach (Johner et al., 2024).

## BB discontinuation

Distinct from the question of long-term continuation, the safety of withdrawing established BB therapy represents a separate clinical scenario. For patients currently on BB therapy, some studies have assessed the safety of discontinuation. A target trial emulation suggests that discontinuing BBs 12 months after ACS is safe in patients with LVEF ≥ 40%, with no increase in MACE risk (Johner et al., 2025). However, in the ABYSS trial, which specifically evaluated treatment withdrawal in patients with a history of MI, interruption of long-term BB treatment failed to demonstrate non-inferiority (Silvain et al., 2024).

Discontinuation of BBs seems to improve quality of life (QoL) by eliminating side effects. However, in the REDUCE-AMI substudy, among patients with preserved LVEF after acute MI, self-reported QoL and wellbeing did not differ significantly between BB and no-BB groups (Mars et al., 2025). Similarly, ABYSS found no QoL improvement with discontinuation (Silvain et al., 2024). Beyond direct risk-benefit considerations, discontinuation decisions should be considered within post-MI polypharmacy realities. An analysis of 90,869 post-MI patients revealed that non-adherence to ACEIs/ARBs and/or statins was associated with increased mortality, whereas BBs showed limited additional benefit (Korhonen et al., 2017). These findings suggest that prioritizing therapies with established efficacy may optimize medication burden and potentially improve overall clinical outcomes.

For post-MI patients with preserved LVEF and no alternative indication, contemporary evidence suggests that discontinuation may be safe and reasonable, though further validation is required. The neutral QoL data relieve clinicians of the need to demonstrate symptomatic benefit to justify discontinuation. For adherence, eliminating medications without proven benefit may be obligatory to optimize therapies.

## EF-stratified framework

Based on accumulating evidence, we propose an EF-stratified framework for BB therapy after MI. For patients with HFrEF (LVEF < 40%), BB initiation and optimization remain mandatory, supported by robust evidence from foundational HF trials (Packer et al., 1996; Packer et al., 2001; Packer et al., 2002; CIBIS-II Investigators and Committees, 1999; MERIT-HF Study Group, 1999). For patients with mildly reduced LVEF (40%–49%), emerging meta-analytic evidence demonstrating a 25% relative risk reduction supports upgrading the recommendation to “should be considered” (Rossello et al., 2025). For patients with preserved LVEF (≥50%) without other compelling indications (e.g., angina, atrial fibrillation requiring rate control), contemporary RCTs have demonstrated neutral effects on hard endpoints (Yndigegn et al., 2024; Ibanez et al., 2025; Munkhaugen et al., 2025), suggesting that routine use for secondary prevention alone may not be supported by current evidence. For patients already receiving BB therapy in this population, a structured approach of reassessment, shared decision-making, and consideration of discontinuation is reasonable.

## Limitations and directions

Several limitations should be considered. First, the open-label design of all three major trials may introduce detection bias and performance bias, while treatment crossover (e.g., 27.9% BB use at 48 months in the REBOOT control arm) may have diluted treatment effects. Second, lower-than-anticipated event rates limited statistical power to detect modest benefits. Furthermore, the enrolled populations predominantly comprised patients aged < 75 years who achieved successful revascularization with low comorbidity burden; thus, extrapolation to elderly, frail, or incompletely revascularized patients requires caution.

Several key questions deserve future investigation: the optimal BB duration in patients with mildly reduced LVEF (40%–49%) patients and whether discontinuation is appropriate after LVEF recovery; whether specific BB subtypes confer differential benefits in the contemporary era; and whether high-risk subgroups within the preserved LVEF population (e.g., large anterior MI, elevated arrhythmic risk) may still derive benefit from BB therapy. Addressing these gaps will require dedicated RCTs with longer follow-up, and inclusion of higher-risk patient subgroups.

## Conclusion

Contemporary evidence has reshaped our understanding of BB therapy after MI in patients without HFrEF. In those with preserved LVEF (≥50%), routine BB therapy has not been shown to reduce the composite of death, MI, or HF in contemporary trials. Conversely, patients with mildly reduced LVEF (40%–49%) appear to derive practical benefit, with a 25% relative risk reduction in the composite endpoint. These findings support an EF-stratified approach: BB therapy remains indicated for LVEF 40%–49% based on emerging meta-analytic data, whereas routine prescription may no longer be warranted for LVEF ≥ 50% in the absence of other indications. For those with preserved LVEF already receiving BBs, reassessment and consideration of discontinuation may be reasonable. This paradigm shift from universal to individualized therapy aligns with contemporary guideline evolution and reflects the diminished therapeutic yield of BB in the era of primary PCI and comprehensive GDMT.

## Data Availability

The original contributions presented in the study are included in the article/Supplementary Material, further inquiries can be directed to the corresponding author.
