# Supplementary material for: Beta-blockers after myocardial infarction with preserved or mildly reduced ejection fraction: existing evidence, knowledge gaps, and an EF-stratified framework
Source: Front Pharmacol. 2026 Apr 21;17:1821921. doi: 10.3389/fphar.2026.1821921 (PMC13139080; doi:10.3389/fphar.2026.1821921)
Supplement: Supplementary file 1 [file Table1.docx]

# SUPPLEMENTARY MATERIALS

**Tables**

**Table S1 Comparison of Clinical Context Between Pre-Modern and Contemporary Post-MI Management**

**Table S2 Clinical end point presentations with beta-blocker therapy regimens after acute myocardial infarction: REDUCE-AMI, REBOOT, and BETAMI–DANBLOCK trials**

# Table S1 Comparison of Clinical Context Between Pre-Modern and Contemporary Post-MI Management

| **Feature** | **Pre-Reperfusion Era**  **(Historical Context)** | **Modern Reperfusion Era (Contemporary Context)** |
| --- | --- | --- |
| Primary Reperfusion | Thrombolysis or conservative management | Routine early coronary angiography with primary PCI in most eligible patients |
| Infarct Characteristics | Larger infarct size, more frequent adverse remodeling/structural sequelae (e.g., left ventricular aneurysm) | Smaller infarct size; greater myocardial salvage |
| Adjunctive Pharmacotherapy | Limited (e.g., aspirin, nitrates) | Comprehensive secondary prevention (e.g., DAPT, high-intensity statins, ACEI/ARB); HF therapies when indicated |
| Diagnostic Sensitivity | Lower (CK-MB); missed micro-infarctions | High-sensitivity Troponins; detection of minor myocardial injury |
| Beta-blocker Evidence | Significant benefit: Reduction in all-cause mortality and recurrent MI. | Equivocal Benefit: minimal to no prognostic benefit in preserved EF (≥ 50%) post-MI, with possible EF-dependent signals in mildly reduced EF (40–49%) |
| Patient Phenotype | High risk of electrical instability and mechanical complications | Lower risk of malignant ventricular arrhythmias/acute mechanical complications; higher comorbidity and competing-risk burden |
| Guideline Focus | Universal prescription for secondary prevention | Stratified approach based on EF and comorbidities |

Abbreviations: MI, myocardial infarction; PCI, percutaneous coronary intervention; DAPT, dual antiplatelet therapy; ACEI, angiotensin-converting enzyme inhibitor; ARB, angiotensin II receptor blocker; HF, heart failure; CK-MB, Creatine Kinase-Myocardial Band; EF, ejection fraction.

# **Table S2 Clinical end point presentations with beta-blocker therapy regimens after acute myocardial infarction: REDUCE-AMI, REBOOT, and** BETAMI–DANBLOCK **trials**

| **REDUCE-AMI** | ***Event no./total no. (%)*** | | |  | |
| --- | --- | --- | --- | --- | --- |
| **Efficacy end points** | **BBs** | **No BBs** | | **HR (95% CI)** | ***P*** |
| Primary: Death from any cause or MI | 199/2,508 (7.9) | 208/2,512 (8.3) | | 0.96 (0.79–1.16) | 0.64# |
| Death from any cause | 97/2,508 (3.9) | 103/2,512 (4.1) | | 0.94 (0.71–1.24) | — |
| Death from cardiovascular causes | 38/2,508 (1.5) | 33/2,512 (1.3) | | 1.15 (0.72–1.84) | — |
| MI | 112/2,508 (4.5) | 117/2,512 (4.7) | | 0.96 (0.74–1.24) | — |
| Hospitalization for AF | 27/2,508 (1.1) | 34/2,512 (1.4) | | 0.79 (0.48–1.31) | — |
| Hospitalization for HF | 20/2,508 (0.8) | 22/2,512 (0.9) | | 0.91 (0.50–1.66) | — |
| **Safety end points** | **BBs** | **No BBs** | | **HR (95% CI)** | ***P*** |
| Hospitalization for bradycardia, second- or third-degree AVB, hypotension, syncope, or implantation of a pacemaker | 86/2,508 (3.4) | 80/2,512 (3.2) | | 1.08 (0.79–1.46) | — |
| Hospitalization for asthma or COPD | 15/2,508 (0.6) | 16/2,512 (0.6) | | 0.94 (0.46–1.89) | — |
| Hospitalization for stroke | 36/2,508 (1.4) | 46/2,512 (1.8) | | RMST difference 6.80 days (−7.11 to 20.72) | — |
| **REBOOT** | ***Event no./total no. (%)**** | |  | |  |
| **Efficacy end points** | **BBs** | **No BBs** | | **HR (95% CI)** | ***P*** |
| Primary: Death from any cause, reinfarction, or hospitalization for HF | 316/4207 (7.5) | 307/4231 (7.3) | | 1.04 (0.89–1.22) | 0.63† |
| Death from any cause | 161/4207 (3.8) | 153/4231 (3.6) | | 1.06 (0.85–1.33) | — |
| Reinfarction | 143/4207 (3.4) | 143/4231 (3.4) | | 1.01 (0.80–1.27) | — |
| Hospitalization for HF | 39/4207 (0.9) | 44/4231 (1.0) | | 0.89 (0.58–1.38) | — |
| Death from cardiac causes | 65/4207 (1.5) | 57/4231 (1.3) | | 1.15 (0.81–1.64) | — |
| Sustained ventricular tachycardia | 3/4207 (< 0.1) | 2/4231 (< 0.1) | | 1.52 (0.25–9.08) | — |
| Ventricular fibrillation | 3/4207 (< 0.1) | 5/4231 (0.1) | | 0.61 (0.14–2.53) | — |
| Resuscitated cardiac arrest | 4/4207 (< 0.1) | 4/4231 (< 0.1) | | 1.01 (0.25–4.05) | — |
| Tertiary: Death from cardiac causes, stroke, or MI | 235/4207 (5.6) | 216/4231 (5.1) | | 1.10 (0.91–1.32) | — |
| Unplanned revascularization | 170/4207 (4.0) | 171/4231 (4.0) | | 1.00 (0.81–1.24) |  |
| **Safety end points** | **BBs** | **No BBs** | | **HR (95% CI)** | ***P*** |
| Hospitalization for symptomatic advanced AVB | 7/4207 (0.2) | 6/4231 (0.1) | | 1.18 (0.40–3.50) | — |
| Hospitalization for stroke | 37/4207 (0.9) | 25/4231 (0.6) | | 1.50 (0.90–2.49) | — |
| **BETAMI–DANBLOCK** | ***Event no./total no. (%)*** | | |  |  |
| **Efficacy end points** | **BBs** | **No BBs** | | **HR (95% CI)** | ***P*** |
| Primary: Composite of death from any cause, MI, unplanned coronary revascularization, ischemic stroke, HF, or MVAs | 394/2,783 (14.2) | 454/2,791 (16.3) | | 0.85 (0.75–0.98) | 0.03§ |
| Death from any cause | 118/2,783 (4.2) | 124/2,791 (4.4) | | 0.94 (0.73–1.21) | — |
| MI | 138/2,783 (5.0) | 186/2,791 (6.7) | | 0.73 (0.59–0.92) | — |
| Unplanned coronary revascularization | 108/2,783 (3.9) | 110/2,791 (3.9) | | 0.99 (0.76–1.29) | — |
| Ischemic stroke | 45/2,783 (1.6) | 35/2,791 (1.3) | | 1.30 (0.84–2.03) | — |
| HF | 42/2,783 (1.5) | 52/2,791 (1.9) | | 0.78 (0.52–1.18) | — |
| MVAs | 15/2,783 (0.5) | 18/2,791 (0.6) | | 0.82 (0.42–1.64) | — |
| Implantation of a pacemaker or second-/third-degree AVB | 49/2,783 (1.8) | 49/2,791 (1.8) | | 1.00 (0.67–1.49) | — |
| **Safety end points** | **BBs** | **No BBs** | | **HR (95% CI)** | ***P*** |
| Composite of death from any cause, MI, HF, or MVA at 30 days | 21/2,783 (0.8) | 32/2,791 (1.1) | | — | — |

Abbreviations: BB, beta-blocker; HR, hazard ratio; CI, confidence interval; MI, myocardial infarction, AF, atrial fibrillation; HF, heart failure; AVB, atrioventricular block; COPD, chronic obstructive pulmonary disease; RMST, restricted mean survival time; MVA, malignant ventricular arrhythmia.

# REDUCE-AMI: Hazard ratios were estimated with Cox proportional-hazards regression; the P value is reported for the primary end point (as specified in the trial).

† REBOOT trial: The P value for the primary end point was calculated with the use of a log-rank test.

***** REBOOT trial: Values in parentheses are percentages (events/total) for the ITT population in this table; in the original trial report, values in parentheses are event rates per 1000 patient-years.

§ BETAMI–DANBLOCK: P = 0.03 for the primary end point (beta-blocker vs no beta-blocker).
